# A General Route for Growing Metal Sulfides onto Graphene Oxide and Exfoliated Graphite Oxide

**DOI:** 10.3390/nano7090245

**Published:** 2017-08-31

**Authors:** Joana L. Lopes, Ana C. Estrada, Sara Fateixa, Marta Ferro, Tito Trindade

**Affiliations:** Department of Chemistry, CICECO—Aveiro Institute of Materials, University of Aveiro, Campus de Santiago, 3810-193 Aveiro, Portugal; jllopes@ua.pt (J.L.L.); ana.estrada@ua.pt (A.C.E.); sarafateixa@ua.pt (S.F.); marta.ferro@ua.pt (M.F.)

**Keywords:** graphene oxide, metal sulfide nanocrystals, Raman spectroscopy

## Abstract

Graphene-based materials are elective materials for a number of technologies due to their unique properties. Also, semiconductor nanocrystals have been extensively explored due to their size-dependent properties that make them useful for several applications. By coupling both types of materials, new applications are envisaged that explore the synergistic properties in such hybrid nanostructures. This research reports a general wet chemistry method to prepare graphene oxide (GO) sheets decorated with nanophases of semiconductor metal sulfides. This method allows the in situ growth of metal sulfides onto GO by using metal dialkyldithiocarbamate complexes as single-molecule precursors. In particular, the role of GO as heterogeneous substrate for the growth of semiconductor nanocrystals was investigated by using Raman spectroscopic and imaging methods. The method was further extended to other graphene-based materials, which are easily prepared in a larger scale, such as exfoliated graphite oxide (EGO).

## 1. Introduction

Graphene is a one-atom-thick layer of sp^2^-bonded carbon atoms arranged in a two-dimensional (2D) honeycomb lattice structure [1]. Due to their electronic, thermal, and mechanical properties, graphene is of great interest in numerous applications, such as water treatment, gas separation [2], catalysis [3], electronics [4], and energy storage in batteries and supercapacitors [5,6]. For certain applications there has been more interest to explore the chemistry of graphene oxide (GO), which can be regarded as a single-layer of graphite oxide. In this case, the presence of structural defects due to oxygen functional groups bound to the 2D carbon monolayer has a detrimental effect on the electronic behavior of pristine graphene. Nevertheless, these oxygen moieties confer to GO layers a hydrophilic character, which makes this material particularly useful for subsequent surface chemical modifications and water compatible applications. An interesting example is the development of hybrid nanostructures that combine GO sheets as the chemical platforms to attach other distinct functional nanophases.

Semiconductor nanocrystals (NC) are important nanomaterials whose interest to associate to GO substrates relies in some extent on the exploitation of synergistic effects by coupling both components. For example, nanosized metal sulfides have attracted great attention because of their size-tunable optical properties [7]. The hydrophilic nature of GO allows for the preparation of colloidal stable suspensions that favor the synthesis in situ of such metal sulfides onto GO surfaces. Some synthetic strategies have been developed for the decoration of GO with semiconductor nanocrystals such as CdS, Bi_2_S_3_, and PbS [8,9,10,11,12,13,14,15,16,17,18,19,20,21,22,23]. Unlike CdS, which has been extensively investigated in association to GO [8,9,10,11,12,13,14,15,16,17,18,19,20], just a few reports were found involving other sulfides such as Bi_2_S_3_, and PbS [21,22,23]. Gao et al. described GO/CdS composites obtained by a two-phase mixing method [18,24]. The preparation of GO nanosheets decorated with CdS nanorods by the pyrolysis of cadmium precursors has been described by Zhang et al. [13] There are several reports of solvothermal routes for the production of GO/metal sulfides [9,12,14,17,19,20]. GO surfaces have been decorated with PbS into a semi-core/shell structure using a solution-based hot-injection method [22].

There has been great interest in using GO as a scaffold to develop visible-light photocatalysts, because it is relatively inexpensive, environmentally friendly, and can enhance the semiconductor photocatalytic properties by extending the light absorption range and improving charge carrier separation and transport [11,14,15,17,18,20,25,26]. Thus far, however, it has not been reported a single-source method for the production of metal sulfides onto GO, with potential for up-scale production of nanocomposite photocatalysts. This gap has been bridged in this study by describing the use of metal alkyldithiocarbamate as precursors to metal sulfides generated in situ in the presence of GO. This research follows our interest in exploring this chemical route by using chemically and structurally distinct substrates, such as amorphous SiO_2_ nanoparticles and carbon nanotubes [27,28]. The simplicity of this method relies on the use of single-molecule precursors to produce the required nanocomposites without segregated phases. This is possible without adding additional sources of sulfur to the reacting system, such as thiourea [10,14,17] or CS_2_ [15]. Also, the method does not require extra polymeric linkers to anchor the metal sulfides to the substrates, such as PVP (polyvinylpyrrolidone) or PAA (polyacrylic acid) [9,14,16]. Hence, this is a general and alternative method to produce several metal sulfides onto GO sheets, at temperatures typically less than 100 °C and relatively short reaction times (less than 1 h). Furthermore, the method reported here is easily applied to other graphene based materials, including less expensive substrates, as illustrated here, by using suspensions containing exfoliated graphite oxide.

## 2. Results and Discussion

**Figure 1:**
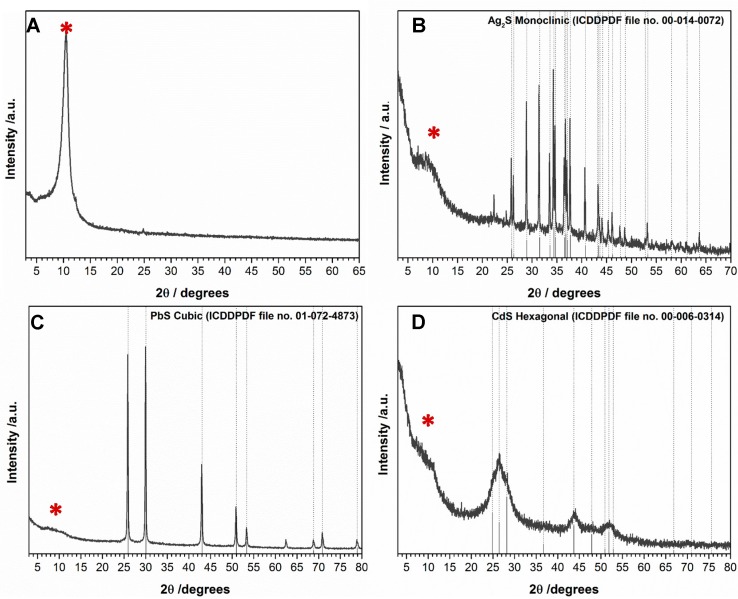
XRD patterns for (**A**) GO; (**B**) GO/Ag_2_S; (**C**) GO/PbS and (**D**) GO/CdS nanostructures. The vertical bars correspond to the diffractions peaks attributed to the respective metal sulfide. The diffraction peaks of GO are marked with *.

The hybrid nanostructures were obtained after generating the metal sulfide nanophases in situ, in the presence of GO sheets, following the reflux of ethanolic suspensions containing a metal alkyldithiocarbamate complex. Figure 1 shows the powder X-ray diffraction (XRD) patterns of GO, and the GO based nanocomposites is reported here. As expected, the GO sample shows powder XRD patterns dominated by a strong peak centered at 2θ = 10.3° corresponding to the 001 plane and to a 0.86 nm interlayer distance [29]. Therefore, we estimate that the GO sample is composed of seven over stacked layers, as deduced from peak broadening and by applying the Debye-Scherrer equation [30]. The intensity of the diffraction peak at 10.3° decreases significantly after decorating the GO with distinct metal sulfide nanophases (Figure 1). It has been reported that the attachment of nanoparticles onto GO may prevent the restacking of suspended graphene sheets, which might explain these observations [31,32]. In addition, the higher crystallinity of the metal sulfides in relation to the GO, thus resulting in sharper and more intense reflection peaks for the latter (Figure S1), can also contribute for these observations. Nonetheless, all diffraction peaks for the hybrid nanostructures were ascribed to the Bragg reflections of the respective metal sulfide, which are in good agreement with the Ag_2_S monoclinic phase (International Centre for Diffraction Data- Powder Diffraction File (ICDDPDF) No. 00-014-0072), CdS hexagonal phase (ICDDPDF No. 00-006-0314) and PbS cubic phase (ICDDPDF No. 01-072-4873), which clearly confirms the presence of the metal sulfides on GO (Figure 1). Note that the peak broadening observed for the CdS sample has been extensively reported as consequence of nanocrystalline diffracting domains [27]. In accordance with the XRD analysis, the energy dispersive X-ray spectroscopy (EDX) measurements performed on all hybrid nanostructures showed the peaks for S and the metal (Ag, Pb, or Cd) of the respective metal sulfide phase. The Cu, C and O peaks observed in the spectrum are attributed to the Cu grid and graphene substrate.

**Figure 2:**
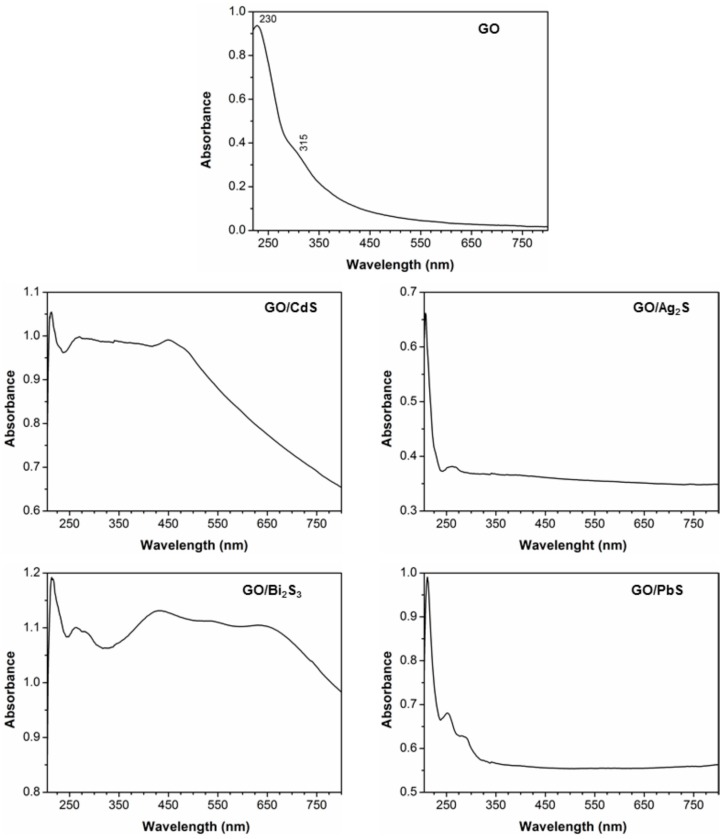
UV/Vis absorption spectra of ethanolic suspensions (0.05 mg/mL) of hybrid carbon nanostructures as indicated in the labels. For comparison, the UV/Vis spectrum of a graphene oxide suspension is also shown.

The optical absorption spectra of the hybrid nanostructures (ethanolic suspensions) reported here are shown in Figure 2. For comparative purposes, the spectra of ethanolic suspensions containing neat GO flakes are also shown. These samples appear macroscopically uniform and the respective digital photographs (Figure S2) show colloidal stable suspensions with distinct colors depending on the metal sulfide obtained. The ultraviolet-visible (UV/VIS) spectrum of GO exhibits an absorption band peaked at 230 nm corresponding to π → π* electronic transitions in the aromatic C–C bonds, and a shoulder at 315 nm ascribed to n → π* transitions in the oxygen functional groups [33]. After growing the metal sulfide phases onto GO sheets, the absorption band at 230 nm for the π-plasmon in the graphitic network was blueshifted to lower wavelength, suggesting the strong coupling between GO sheets and metal sulfide phases [10,34,35,36]. In fact, it has been reported that an increase in the number of functional groups or defects in the graphene structure causes the decrease of the π-conjugated domain size, thus resulting in the shift to lower wavelength of the bands associated to the polyaromatic structure [34,37]. Although the presence of oxygen moieties in GO causes disruption of the graphene delocalized system, we interpret similarly the blueshift observed in the π → π* optical spectrum of the GO/metal sulfide due to the contribution of metal sulfide-GO interactions. Also, note that for all of the hybrids, the absorption band at 315 nm, corresponding to the C–O linkages, was shifted to lower values.

Figure 2 indicates that all of the nanocomposites absorb stronger in the visible region of the spectrum (390–700 nm). These results confirm the presence of the metal sulfides in the GO sheets, as expected by taking into consideration the reported band gap energies for the respective bulk semiconductor: Ag_2_S (Eg = 1.0 eV, λ_onset_ = 1240 nm ), PbS (Eg = 0.41 eV, λ_onset_ = 3024 nm), CdS (Eg = 2.42 eV, λ_onset_ = 512 nm), and Bi_2_S_3_ (Eg=1.3 eV, λ_onset_ = 953 nm). Additionally, the CdS/GO sample shows an absorption peak at 460 nm, which we suggest can be indicative of the presence of small nanocrystallites showing quantum-sized behavior. These smaller nanocrystallites can be present in the GO substrate, namely by clustering into larger nanostructures but retaining their crystallite size domain [28], as will be shown below. This size-dependent optical behavior has been well documented for diverse semiconductors with particle dimensions comparable or below the corresponding Bohr radius of the exciton in the macrocrystalline material [38,39]. The GO/PbS nanocomposite shows a broad absorption band that extends to the infrared region, whereas the Bi_2_S_3_ hybrid material shows two absorption peaks in the visible, at 430 nm and 650 nm, which might be due to the presence of distinctly sized crystallites.

**Figure 3:**
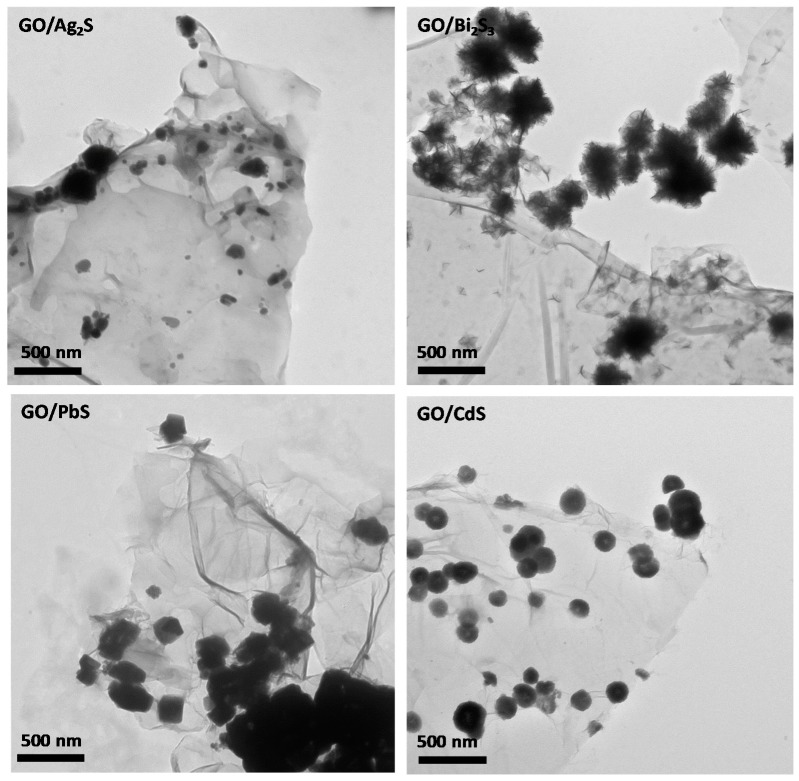
TEM images of graphene oxide hybrid nanostructures containing the metal sulfide nanophases indicated in the labels.

**Figure 4:**
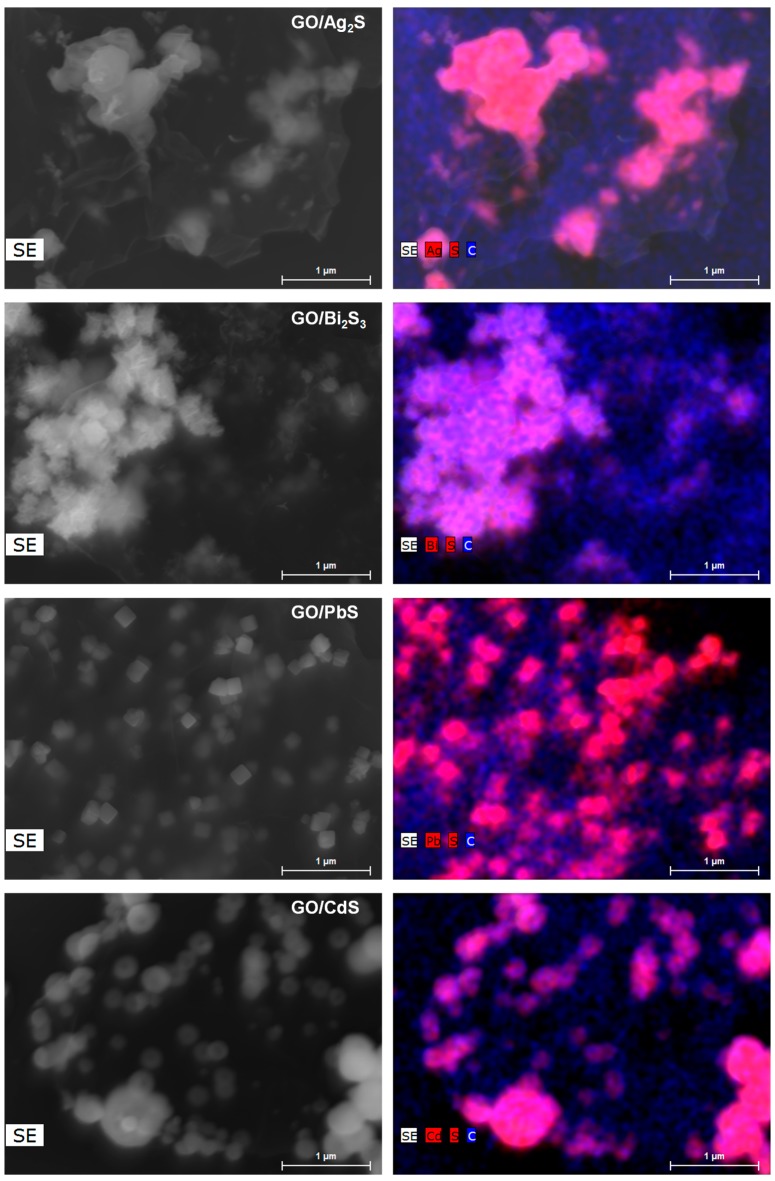
SEM images (**left** panel) and EDX mappings (**right** panel) of graphene oxide hybrid nanostructures containing metal sulfide nanophases.

**Figure 5:**
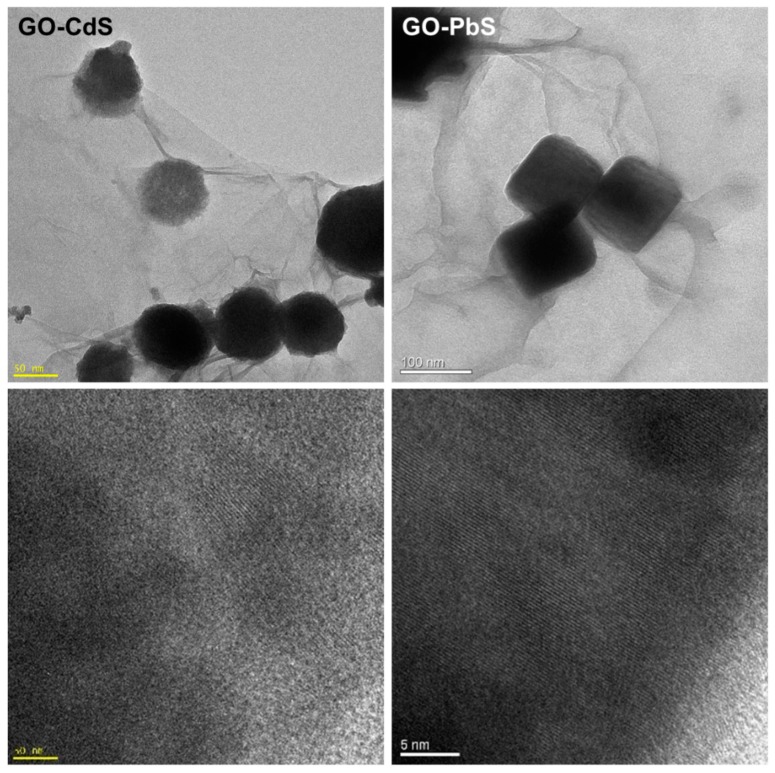
Graphene oxide (GO) sheets decorated with CdS and PbS nanophases: TEM images (**top**) and corresponding HRTEM images (**bottom**).

Figure 3 and Figure 4 show, respectively, the transmission electron microscopy (TEM) and scanning electron microcopy (SEM) images of the hybrid samples. The TEM images display a number of darker spots dispersed over a background composed of GO sheets. These images prove that the metal sulfides are present in these samples as nanophases decorating the GO sheets. The nanophases distributed over the GO surfaces retain their morphological identity although size polydispersity occurs to some extent. The SEM images are in line with these findings and the respective EDX mappings (Figure 4) confirm the presence of the respective metal sulfide elements onto the GO substrates. The inspection of several samples did not show evidence for the presence of semiconductor particles scattered out of the GO substrate. Indeed, further experiments involving the sonication of hybrid nanostructures suspensions did not result in a significant number of free metal sulfide particles. The inspection of the high-resolution TEM images confirms the presence of crystalline particles onto GO, as illustrated in Figure 5, for PbS and CdS, by the presence of the lattice fringes of the metal sulfides. These results are consistent with a surface mediated mechanism in which the metal sulfide particulates nucleate and grow on the GO sheets. The metal sulfide is formed due to the degradation of the metal alkyldithiocarbamate precursor, which is a single-source for sulfide species and metal ions, according to a mechanism previously proposed [40].

**Figure 6:**
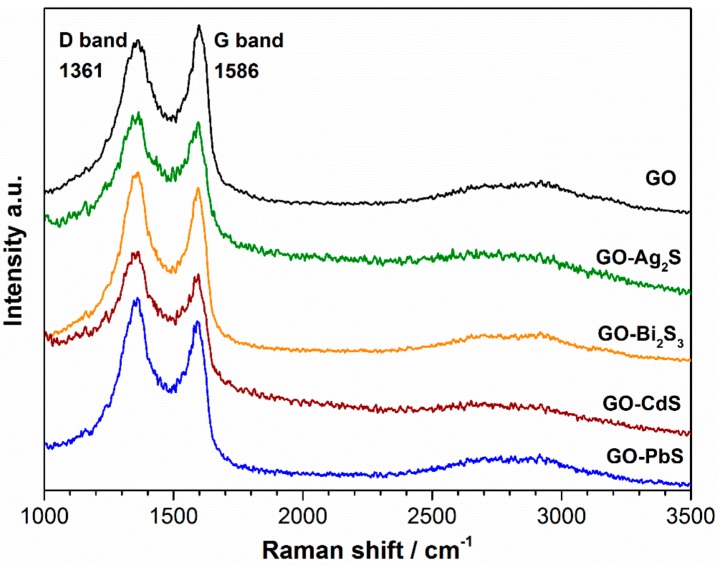
Raman spectra of graphene oxide and derived hybrids having the indicated metal sulfides (1000–3500 cm^−1^ region).

Raman spectroscopic methods have been successfully employed in the characterization of a number of carbon nanostructures, including graphene-based materials [41]. The results discussed above suggest that the metal sulfides grown over the GO sheets have an impact on the structural features of the carbon layers. As such, we have decided to investigate in more detail these effects by using Raman methods, in particular, by taking into consideration that conjugated carbon-carbon bonds in graphene materials result into distinct Raman signatures. Figure 6 shows the Raman spectra for the hybrid nanostructures and pure GO, used as substrate. The two bands observed are characteristic of graphitic materials, corresponding to the vibration of bonded sp^2^-carbons of the two-dimensional hexagonal lattice (G band at 1580 cm^−1^) and to sp^3^-carbon containing moieties that act as defects introduced in the graphene lattice (D band at 1350 cm^−1^) [41,42,43,44,45,46]. Table 1 summarizes relevant Raman data collected from the spectroscopic measurements performed on the GO and hybrid nanostructures. In comparison to the Raman spectrum of GO, the G, and D bands in the Raman spectrum of each hybrid nanostructure are slightly shifted to lower wavenumbers. We attribute these shifts to structural modifications occurring in the GO sheets during the synthesis, which are eventually mediated by interactions with the metal sulfide. Although using distinct materials, Zhu et al. have reported ZnO/graphene composites whose Raman spectra showed shifts to lower wavenumbers for the D band and shifts to higher wavenumbers for the G band [43]. Here, both of the G and D bands in the Raman spectrum of each nanocomposite are shifted to lower wavenumbers. These shifts for lower wavenumbers can also be associated to the formation of reduced GO [46]. However, in the case of reduced GO formation, we would also expect an increase of the (D-band/G-band) intensity ratio [46]. In fact, Table 1 indicates a slight increase of the Raman intensities ratio (D-band/G-band), suggesting an increase of the number of defects in the nanocomposites, as compared to pristine GO. The origin of such defects could be traced to the treatment of the GO flakes during the synthesis. The broad band at ~2680 cm^−1^ (2D-band) is the second order of the D-band, that results from a two phonon lattice vibrational process, but that does not need to be activated by the proximity of defects [45]. Unlike monolayer graphene, whose Raman spectrum shows a sharp 2D band with stronger intensity than the G band, the materials reported here show broad and less intense Raman 2D-bands as expected for a multilayered GO [47]. 

**Figure 7:**
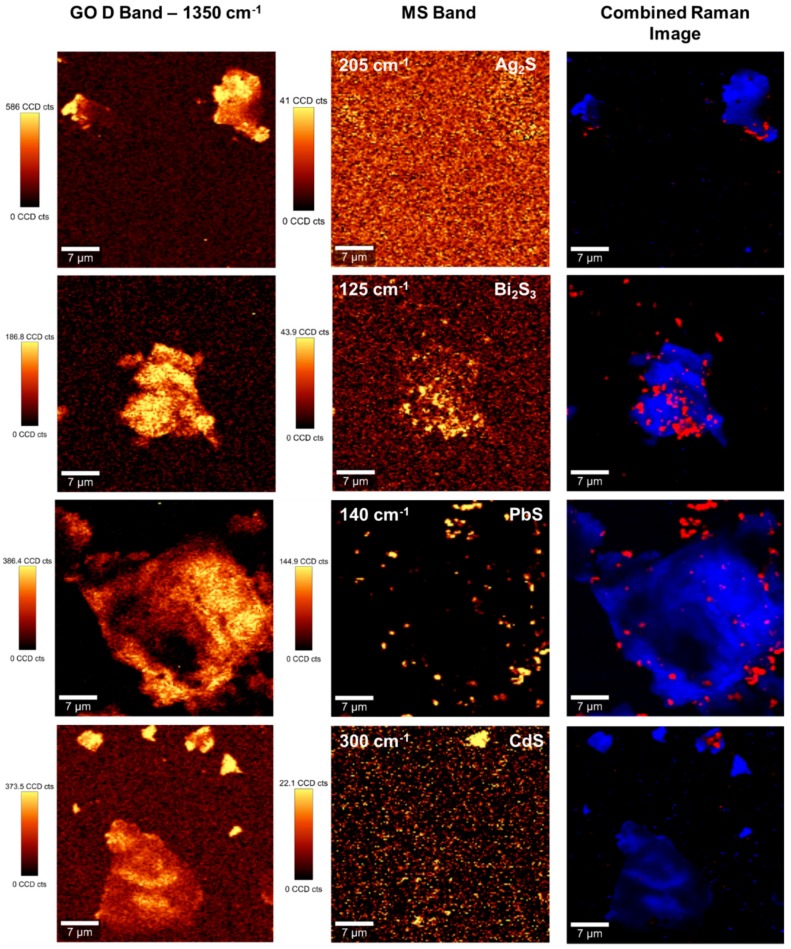
Raman images of GO decorated with metal sulfides (as indicated in the **middle** panel images). The Raman images were obtained by using the integrated intensity of the Raman band of GO at 1350 cm^−1^ (**left** panel) and the characteristic Raman band of the metal sulfide as indicated (**middle** panel)—excitation line at 532 nm, 1 mW laser power, 150 × 150 points per grid in a 40 × 40 μm area. The vertical bar shows the color profile in the Raman images, with the relative intensity scale. The combined Raman images of the GO substrate and metal sulfides are also shown (**right** panel).

**Figure 8:**
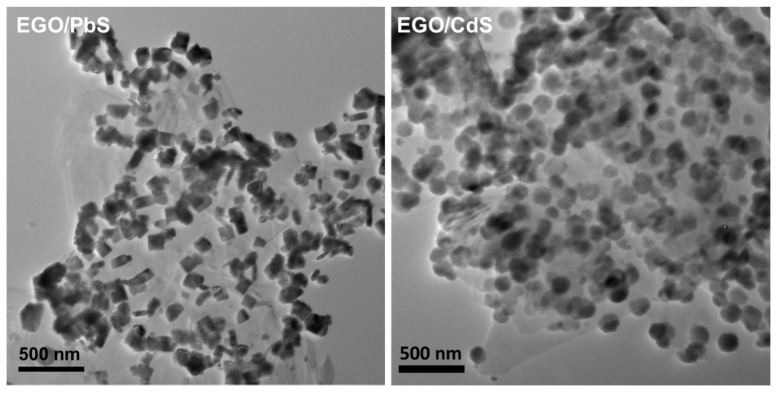
Transmission electron microscopy (TEM) images of exfoliated graphite oxide (EGO) decorated with PbS and CdS nanocrystals.

Raman mapping has been a less exploited tool in the characterization of decorated graphene type materials, such as the hybrid nanostructures reported in this research. Taking advantage of the Raman signature assigned to the presence of sp^3^-carbon defects, at about 1350 cm^−1^, we have performed Raman imaging studies for neat GO and metal sulfide doped GO. These screening experiments show micrometric regions enriched in defects distributed over the GO sheets (Figure 7—left panel). On the other hand, the metal sulfides deposited on GO show characteristic Raman shifts at wavenumbers lower than the G band, as shown in Figure S3. These Raman bands are characteristic of each metal sulfide and were used for screening the regions of GO coated with the respective semiconductor nanophase (Figure 7—middle panel). The combined Raman images (Figure 7—right panel) for the GO sheets and the corresponding doped GO samples, give a fair agreement with the assertion that the metal sulfides are mainly located in GO regions enriched in defects. The Raman mapping of the hybrid samples also corroborate the presence of metal sulfide nanophases distributed over the GO sheets, as discussed above for the TEM analysis. The method described here for decorating GO sheets can be applied to other carbon materials or by exploring other thermal treatment methods [27,48,49]. For example, it would be of great interest to apply this synthesis using less expensive carbon substrates and which also allow large-scale production. As an illustrative case, we have applied a similar synthetic methodology but using suspensions of exfoliated graphite oxide (EGO) instead of GO (Figure S4). Figure 8 shows the TEM images for EGO flakes decorated with CdS and PbS nanophases. Although the TEM results suggest that there is a higher population of discrete metal sulfide phases over the surfaces of the EGO hybrid materials, overall the morphological features of such hybrids resemble those of the GO analogues.

## 3. Materials and Methods

### 3.1. Chemicals and Methods

Ethylenediamine (Sigma-Aldrich, St. Louis, MO, USA), graphite (GK, Hauzenberg, Germany), graphene oxide aqueous solution (2.5 mg/mL, Nanocs, New York, NY, USA) dimethylformamide (Carlo Erba, Milan, Italy) were used as received. All other solvents were obtained from commercial sources and used as received or distilled and dried by standard procedures.

The following metal diethyldithiocarbamates were used as single-molecule precursors {M[S_2_CN(C_2_H_5_)_2_]_x_}, where M = Ag, Cd, Bi, and Pb [27]. All of the compounds were prepared by the stoichiometric reaction of Na(S_2_CN(C_2_H_5_)_2_) and the respective metal nitrate, in water. The solid obtained, was thoroughly washed and collected by filtration.

Exfoliated graphite oxide was produced by the sonochemical exfoliation of graphite in a high boiling point solvent (DMF), in accordance with the literature [50]. In a typical experiment, a DMF dispersion of graphite flakes (50 mg/mL) was submitted to a sonochemical reaction during 5 h. The resulting suspension was centrifuged (500 rpm, 45 min) using a Eba 20 Hettich centrifuge, and filtered under reduced pressure. The final powder obtained was left to dry at 40 °C.

### 3.2. Synthesis of Hybrid Carbon Nanostructures

All procedures were performed in a well-ventilated fume cupboard. The GO sheets were decorated with metal sulfides by the addition of ethylenediamine (9 mmol) to a dry ethanol suspension of GO (20 mg, 25 mL) containing the metal precursor (58 μmol). The mixture was then stirred at reflux until a color change of the reaction mixture was clearly observed. The hybrid nanostructures were then collected by centrifugation (6000 rpm, 15 min) using an Eba 20 Hettich centrifuge and thoroughly washed with ethanol. Finally, the powders were dried at room temperature and kept under N_2_.

### 3.3. Instrumentation

The UV/Vis spectra were recorded using a Jasco V-560 spectrometer (Jasco Inc., Easton, MD, USA). The X-ray power diffraction (XRD) data were collected using a PANanalytical Empyrean X-ray diffractometer (PANanalytical, Almelo, The Netherlands) equipped with Cu-K*α* monochromatic radiation source at 45 kV/40 mA. The samples were prepared by the deposition of aliquots of the ethanolic suspensions in hybrid nanostructures on a silicon holder. XRD patterns of the GO-MS nanocomposites with the internal reference (Si) were recorded similarly. For transmission electron microscopy (TEM) analysis, a drop of the suspension under analysis was placed on a carbon-coated Cu grid and the solvent was left to evaporate at room temperature. TEM was performed using a transmission electron microscope Hitachi H-9000 (Hitachi, Tokyo, Japan) operating at 300 kV. The high-resolution TEM images were acquired by using a JEOL 2200FS HR-TEM instrument (JEOL, Tokyo, Japan). SEM analysis was performed using a scanning electron microscope Hitachi SU-70 (Hitachi, Tokyo, Japan) operating at an accelerating voltage of 15 kV. Energy dispersive X-ray spectroscopy (EDX) analysis was performed using Bruker Esprit (Berlin, Germany). Raman spectral imaging was performed in a combined Raman-AFM confocal microscope WITec alpha300 RAS+ (WITec, Ulm, Germany). A Nd:YAG laser operating at 532 nm was used as excitation source with the power set at 1 mW. The Raman confocal microscope was calibrated by acquiring the spectrum of a silicon wafer (532 nm laser source, 0.5 s, 10 acquisitions, 40 mW laser power). The Gaussian function was fitted to the Raman band at 521 cm^−1^, and an error of 0.08 cm^−1^ was obtained. The intensity values and associated errors of the Raman bands of pristine GO and the nanocomposites were obtained by multi-peaks fitting using the Gaussian function in Origin 8. Raman imaging experiments were performed by raster-scanning the laser beam over the samples and accumulating a full Raman spectrum at each pixel. The WITec software, WITec Project 2.0 (WITec, Ulm, Germany), was used to create the Raman images. The Raman images showing a color scale have been created using band integrals, in which the value of the absolute area underneath a band (e.g., 1350 cm^−1^) corresponds to color intensity in the scale, shown in the image at the respective pixels. The Raman spectra of the semiconductor and the GO were used as the basis set in the analysis using the software tool of WITec Project, providing the color-coded combined Raman images.

## 4. Conclusions

GO-metal sulfide hybrid nanostructures have been successfully produced by in situ thermal decomposition of the respective metal alkyldithiocarbamato complexes. This one-step method is scalable for producing hybrid GO materials with negligible phase segregation and might be extended to other graphene-based materials. The method produces GO materials decorated with metal sulfides, whose absorption in the visible can be tuned by the deposited semiconducting nanophase. We are currently exploring this approach to trigger photon harvesting in water compatible photocatalysts that perform efficiently in the visible spectral region.

## Figures and Tables

**Table 1:** Raman shifts for G and D bands with the respective intensities ratio.

Sample	Band Positions (cm^−1^)		
	D	G	I_D_/I_G_
**Graphene oxide (GO)**	1366.5 ± 1.2	1595.7 ± 0.7	0.92
**GO/Bi_2_S_3_**	1359.5 ± 1.2	1587.9 ± 0.9	1.05
**GO/Ag_2_S**	1361.9 ± 1.1	1585.3 ± 0.7	1.02
**GO/CdS**	1361.4 ± 0.9	1580.1 ± 0.8	1.07
**GO/PbS**	1360.6 ± 1.1	1581.5 ± 1.0	1.07

## Supplementary Materials

The following are available online at http://www.mdpi.com/2079-4991/7/9/245/s1, Figure S1: Powder XRD patterns of GO/MS hybrid nanostructures, Figure S2: Digital photographs of metal sulfide/GO suspensions, Figure S3: Raman spectra of GO sheets and derived hybrid nanostructures, Figure S4: Powder XRD patterns of EGO/MS hybrid nanostructures.

## References

[1] Georgakilas V., Perman J.A., Tucek J., Zboril R. (2015). Broad family of carbon nanoallotropes: Classification, chemistry, and applications of fullerenes, carbon dots, nanotubes, graphene, nanodiamonds, and combined superstructures. Chem. Rev..

[2] Titirici M.M., White R.J., Brun N., Budarin V.L., Su D.S., del Monte F., Clark J.H., MacLachlan M.J. (2015). Sustainable carbon materials. Chem. Soc. Rev..

[3] Seger B., Kamat P.V. (2009). Electrocatalytically active graphene-platinum nanocomposites. Role of 2-D carbon support in PEM fuel cells. J. Phys. Chem. C.

[4] Yan Q., Huang B., Yu J., Zheng F., Zang J., Wu J., Gu B.L., Liu F., Duan W. (2007). Intrinsic current–voltage characteristics of graphene nanoribbon transistors and effect of edge doping. Nano Lett..

[5] Yoo E.J., Kim J., Hosono E., Zhou H., Kudo T., Honma I. (2008). Large reversible Li storage of graphene nanosheet families for use in rechargeable lithium ion batteries. Nano Lett..

[6] Stoller M.D., Park S., Zhu Y., An J., Ruoff R.S. (2008). Graphene-based ultracapacitors. Nano Lett..

[7] Trindade T., Thomas P.J., Reedijk J., Poeppelmeter K. (2013). Solid-state materials, including ceramics and minerals. Comprehensive Inorganic Chemistry II: From Elements to Applications.

[8] Cao A., Liu Z., Chu S., Wu M., Ye Z., Cai Z., Chang Y., Wang S., Gong Q., Liu Y. (2010). A facile one-step method to produce graphene-CdS quantum dot nanocomposites as promising optoelectronic materials. Adv. Mater..

[9] Wang P., Jiang T., Zhu C., Zhai Y., Wang D., Dong S. (2010). One-step, solvothermal synthesis of graphene-CdS and graphene-ZnS quantum dot nanocomposites and their interesting photovoltaic properties. Nano Res..

[10] Wu J., Bai S., Shen X., Jiang L. (2010). Preparation and characterization of graphene/CdS nanocomposites. Appl. Surf. Sci..

[11] Zhang N., Zhang Y., Pan X., Fu X., Liu S., Xu Y.-J. (2011). Assembly of CdS nanoparticles on the two-dimensional graphene scaffold as visible-light-driven photocatalyst for selective organic transformation under ambient conditions. J. Phys. Chem. C.

[12] Li Q., Guo B., Yu J., Ran J., Zhang B., Yan H., Gong J.R. (2011). Highly efficient visible-light-driven photocatalytic hydrogen production of CdS-cluster-decorated graphene nanosheets. J. Am. Chem. Soc..

[13] Zhang K., Liu X. (2011). One step synthesis and characterization of CdS nanorod/graphene nanosheet composite. Appl. Surf. Sci..

[14] Gao Z., Liu N., Wu D., Tao W., Xu F., Jiang K. (2012). Graphene-CdS composite, synthesis and enhanced photocatalytic activity. Appl. Surf. Sci..

[15] Pan S., Liu X. (2012). CdS–Graphene nanocomposite: Synthesis, adsorption kinetics and high photocatalytic performance under visible light irradiation. New J. Chem..

[16] Narayanam P.K., Singh G., Botcha V.D., Sutar D.S., Talwar S.S., Srinivasa R.S., Major S.S. (2012). Growth of CdS nanocrystallites on graphene oxide Langmuir-Blodgett monolayers. Nanotechnology.

[17] Liu F., Shao X., Wang J., Yang S., Li H., Meng X., Liu X., Wang M. (2013). Solvothermal synthesis of graphene-CdS nanocomposites for highly efficient visible-light photocatalyst. J. Alloys Compd..

[18] Gao P., Liu J., Delai D., Ng W. (2013). Graphene oxide-CdS composite with high photocatalytic degradation and disinfection activities under visible light irradiation. J. Hazard. Mater..

[19] Zhang N., Yang M.Q., Tang Z.R., Xu Y.J. (2013). CdS-graphene nanocomposites as visible light photocatalyst for redox reactions in water: A green route for selective transformation and environmental remediation. J. Catal..

[20] Lü W., Chen J., Wu Y., Duan L., Yang Y., Ge X. (2014). Graphene-enhanced visible-light photocatalysis of large-sized CdS particles for wastewater treatment. Nanoscale Res. Lett..

[21] Li G., Chen X., Gao G. (2014). Bi_2_S3 microspheres grown on graphene sheets as low-cost counter-electrode materials for dye- sensitized solar cells. Nanoscale.

[22] Tavakoli M.M., Aashuri H., Simchi A., Kalytchuk S., Fan Z. (2015). Quasi core/shell lead sulfide/graphene quantum dots for bulk heterojunction solar cells. J. Phys. Chem. C.

[23] Tayyebi A., Tavakoli M.M., Outokesh M., Shafiekhani A., Simchi A. (2015). Supercritical synthesis and characterization of “graphene-PbS quantum dots” composite with enhanced photovoltaic properties. Ind. Eng. Chem. Res..

[24] Gao P., Liu J., Lee S., Zhang T., Sun D.D. (2012). High quality graphene oxide-CdS-Pt nanocomposites for efficient photocatalytic hydrogen evolution. J. Mater. Chem..

[25] Chen F., Jia D., Jin X., Cao Y., Liu A. (2016). A general method for the synthesis of graphene oxide-metal sulfide composites with improved photocatalytic activities. Dyes Pigment..

[26] Ren Z., Zhang J., Xiao F.-X., Xiao G. (2014). Revisiting the construction of graphene-CdS nanocomposites as efficient visible-light-driven photocatalysts for selective organic transformation. J. Mater. Chem. A.

[27] Estrada A.C., Mendoza E., Trindade T. (2014). Decoration of carbon nanostructures with metal sulfides by sonolysis of single-molecule precursors. Eur. J. Inorg. Chem..

[28] Monteiro O.C., Esteves A.C.C., Trindade T. (2002). The synthesis of SiO_2_@CdS nanocomposites using single-molecule precursors. Chem. Mater..

[29] Sharon M., Sharon M. (2015). Graphene: An Introduction to the Fundamentals and Industrial Applications.

[30] Saikia B.K., Boruah R.K., Gogoi P.K. (2009). A X-ray diffraction analysis on graphene layers of assam coal. J. Chem. Sci..

[31] Pham T.A., Choi B.C., Jeong Y.T. (2010). Facile covalent immobilization of cadmium sulfide quantum dots on graphene oxide nanosheets: Preparation, characterization, and optical properties. Nanotechnology.

[32] Wang X., Tian H., Yang Y., Wang H., Wang S., Zheng W., Liu Y. (2012). Reduced graphene oxide/CdS for Efficiently photocatalystic degradation of methylene blue. J. Alloys Compd..

[33] Li D., Müller M.B., Gilje S., Kaner R.B., Wallace G.G. (2008). Processable aqueous dispersions of graphene nanosheets. Nat. Nanotechnol..

[34] Luo J., Cote L.J., Tung V.C., Tan A.T.L., Goins P.E., Wu J., Huang J. (2010). Graphene oxide nanocolloids. J. Am. Chem. Soc..

[35] Yang Y., Liu T. (2011). Fabrication and characterization of graphene oxide/zinc oxide nanorods hybrid. Appl. Surf. Sci..

[36] Wu J., Shen X., Jiang L., Wang K., Chen K. (2010). Solvothermal synthesis and characterization of sandwich-like graphene/ZnO nanocomposites. Appl. Surf. Sci..

[37] Zhang L., Liang J., Huang Y., Ma Y., Wang Y., Chen Y. (2009). Size-controlled synthesis of graphene oxide sheets on a large scale using chemical exfoliation. Carbon.

[38] Brus L. (1991). Quantum crystallites and nonlinear optics. Appl. Phys. A.

[39] Weller H. (1993). Quantized semiconductor particles: A novel state of matter for materials science. Adv. Mater..

[40] Neves M.C., Monteiro O.C., Hempelmann R., Silva A.M.S., Trindade T. (2008). From single-molecule precursors to coupled Ag_2_S/TiO_2_ nanocomposites. Eur. J. Inorg. Chem..

[41] Ferrari A.C., Basko D.M. (2013). Raman spectroscopy as a versatile tool for studying the properties of grapheme. Nat. Nanotechnol..

[42] Dresselhaus M.S., Jorio A., Hofmann M., Dresselhaus G., Saito R. (2010). Perspectives on carbon nanotubes and graphene Raman spectroscopy. Nano Lett..

[43] Xu T., Zhang L., Cheng H., Zhu Y. (2011). Significantly enhanced photocatalytic performance of ZnO via graphene hybridization and the mechanism study. Appl. Catal. B.

[44] Beams R., Cançado L.G., Novotny L. (2015). Raman characterization of defects and dopants in graphene. J. Phys..

[45] Zhang X., Rajaraman B.R.S., Liu H., Ramakrishna S. (2014). Graphene’s potential in materials science and engineering. RSC Adv..

[46] Lambert T.N., Chavez C.A., Hernandez-Sanchez B., Lu P., Bell N.S., Ambrosini A., Friedman T., Boyle T.J., Wheeler D.R., Huber D.L. (2009). Synthesis and characterization of Titania-Graphene nanocomposites. J. Phys. Chem. C.

[47] Berciaud S., Li X., Htoon H., Brus L.E., Doorn S.K., Heinz T.F. (2013). Intrinsic line shape of the raman 2D-mode in freestanding graphene monolayers. Nano Lett..

[48] Paz F.A., Rocha J., Klinowski J., Trindade T., Shi S., Mafra L. (2005). Optimised hydrothermal synthesis of multi-dimensional hybrid coordination polymers containing flexible organic ligands. Prog. Solid State Chem..

[49] Martins N.C.T., Angelo J., Girão A.V., Trindade T., Andrade L., Mendes L.A. (2016). N-doped carbon quantum dots/TiO_2_ composite with improved photocatalytic activity. Appl. Catal. B.

[50] Khan U., O’Neill A., Lotya M., De S., Coleman J.N. (2010). High-concentration solvent exfoliation of graphene. Small.

