# Supplementary material for: A General Route for Growing Metal Sulfides onto Graphene Oxide and Exfoliated Graphite Oxide
Source: Nanomaterials (Basel). 2017 Aug 31;7(9):245. doi: 10.3390/nano7090245 (PMC5618356; doi:10.3390/nano7090245)
Supplement: Supplementary file 1 [file nanomaterials-07-00245-s001.pdf]

Supplementary Materials

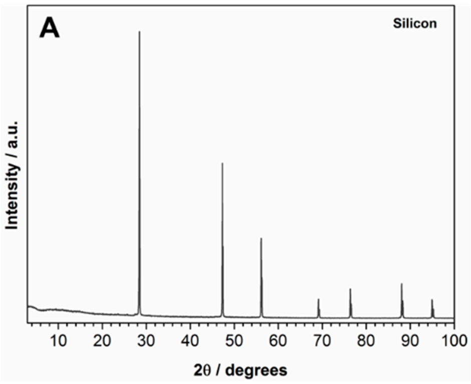

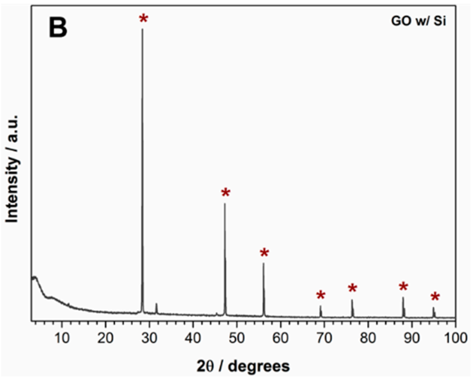

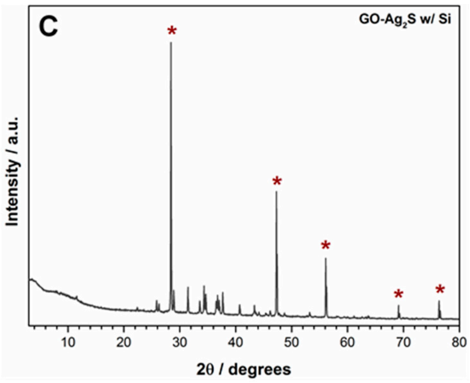

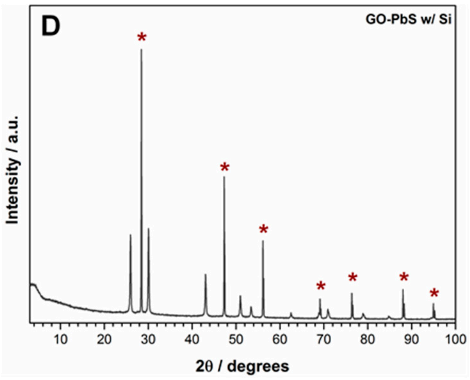

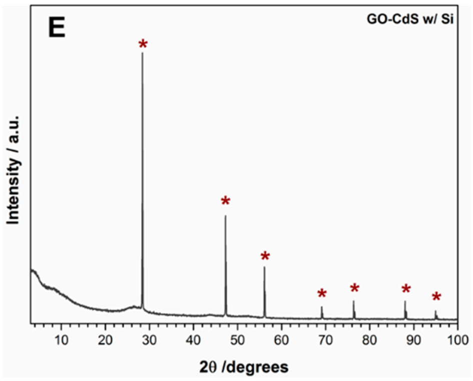

Figure S1. *(A-E) Powder XRD patterns of GO/MS hybrid nanostructures as indicated, with Si as internal reference (marked as *).*

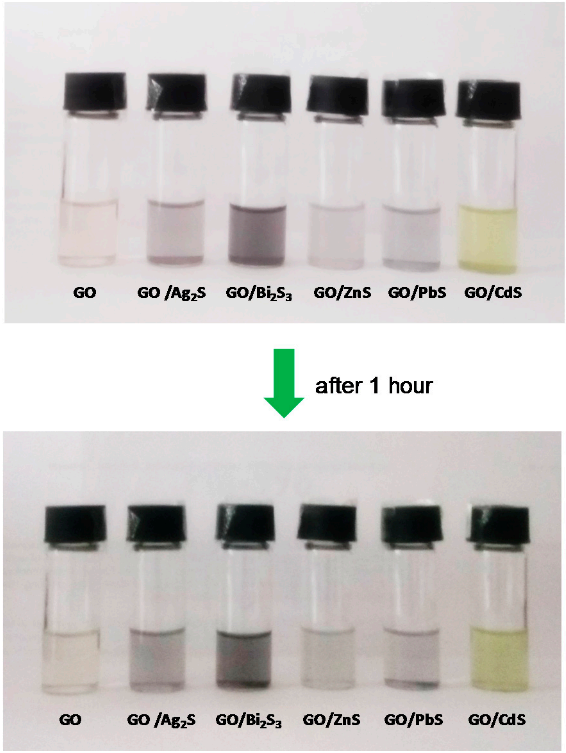

Figure S2*. Ethanolic suspensions of GO/metal sulfide (0.05 mg/ mL) after preparation and 1 h standing.*

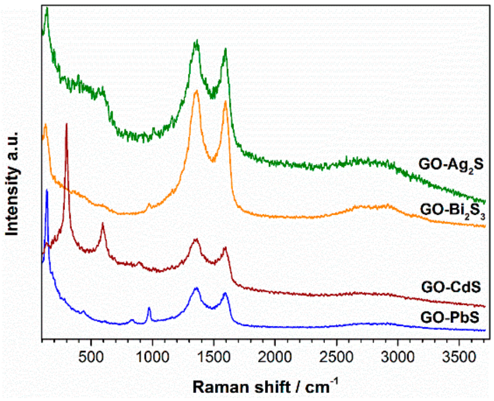

Figure S3. *Raman spectra of GO sheets and derived hybrid nanostructures having the indicated metal sulfide.*

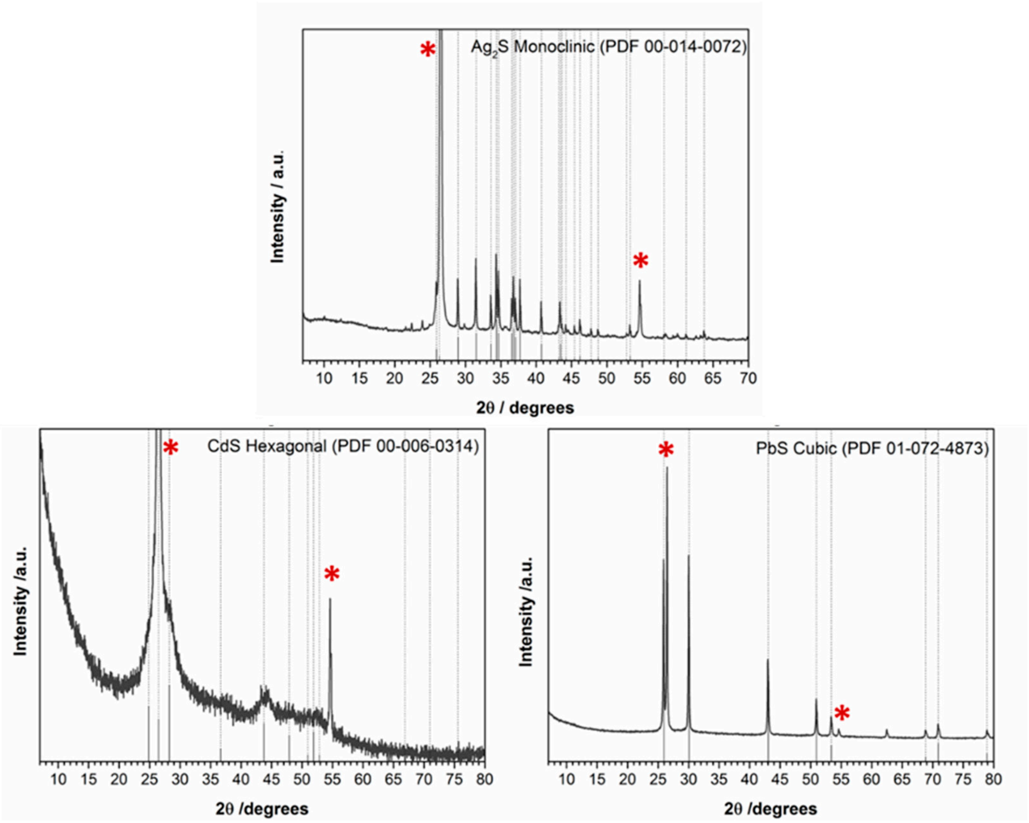

Figure S4. *Powder XRD patterns of EGO/MS hybrid nanostructures. The vertical lines correspond to the standard diffractions peaks attributed to the respective metal sulfide and the diffraction peaks assigned to EGO are marked with **
